# Prognosis of Congenital Anomalies in Conceptions Following *In Vitro* Fertilization: A Multicenter Retrospective Cohort Study in China

**DOI:** 10.3389/fendo.2022.900499

**Published:** 2022-07-14

**Authors:** Jie Bao, Lixue Chen, Yongxiu Hao, Hongping Wu, Xiaojin He, Chuncheng Lu, Xinhua Ji, Jie Qiao, Yuanyuan Wang, Hongbin Chi

**Affiliations:** ^1^ Center for Reproductive Medicine, Department of Obstetrics and Gynecology, Peking University Third Hospital, Beijing, China; ^2^ National Clinical Research Center for Obstetrics and Gynecology, Peking University Third Hospital, Beijing, China; ^3^ Key Laboratory of Assisted Reproduction, Peking University, Ministry of Education, Beijing, China; ^4^ Beijing Key Laboratory of Reproductive Endocrinology and Assisted Reproductive Technology, Beijing, China; ^5^ Center for Reproductive Medicine, The First Affiliated Hospital of Anhui Medical University, Hefei, China; ^6^ School of Public Health, Nanjing Medical University, Nanjing, China; ^7^ International Peace Maternity and Child Health Hospital, School of Medicine, Shanghai Jiao Tong University, Shanghai, China

**Keywords:** congenital anomalies, *in vitro* fertilization, fertilization failure, pregnancy outcome, prognosis

## Abstract

**Background:**

Conceptions following *in vitro* fertilization (IVF) or intracytoplasmic sperm injection (ICSI) have an increased risk of congenital anomalies. Few studies have explored the prognosis of fetuses with congenital anomalies. This study aimed to investigate the prevalence and prognosis of congenital anomalies in IVF/ICSI pregnancies, and to analyze the influencing factors contributing to poor prognosis.

**Methods:**

In this multicenter retrospective cohort study, we followed 405,473 embryo transfer cycles at 15 reproductive centers between January 2010 and December 2019 and enrolled 2,006 intrauterine pregnancies with congenital anomalies. The relatively positive prognosis group with one or more live births and neonatal survival for more than 7 days was compared with the poor prognosis group with poorer outcomes.

**Results:**

Among the 168,270 ongoing intrauterine pregnancy cycles, the prevalence of congenital anomalies was 1.19%, wherein the malformation rates of cycles with late abortion and delivery were 2.37% (716/30,202) and 0.93% (1,290/138,068), respectively. Among all IVF/ICSI cycles with congenital anomalies, the relatively positive prognosis rate was 61.39%. Moreover, the fertilization failure rate (2 pro-nuclei rate < 25%) in the poor prognosis group was significantly higher than that in the relatively positive prognosis group (10.89% vs. 5.09%, *p* < 0.001). Multivariate logistic regression analysis revealed no significant differences in the relatively positive prognosis rate among the various IVF/ICSI protocols. The relatively positive prognosis rate of fertilization failure cycles was 0.180 times that of normal fertilization cycles.

**Conclusion:**

Poor fertilization rates during IVF/ICSI treatments are more likely to have poor prognosis in fetuses or neonates with congenital anomalies, and obstetric management should be strengthened in pregnant women, with which pregnant women should be recommended to strengthen obstetric management.

## Introduction

Over the decades, with the continuous development and social acceptance of assisted reproductive technologies (ARTs), an increasing number of infertile couples have conceived by undergoing *in vitro* fertilization (IVF) or intracytoplasmic sperm injection (ICSI). In European countries, approximately 2% of infants are born annually through ART (1). In Beijing, the rate of births attributed to ART has reached 1.4%, which is close to the estimated rate of ART conceptions in China (1%–2%) (2). Although this technique primarily aims to improve pregnancy outcomes in infertile couples, there remains widespread concern and controversy about whether IVF/ICSI increases the incidence of adverse obstetric and perinatal outcomes due to its unnatural *in vitro* procedures.

Congenital anomalies are a leading cause of fetal or neonatal death in the perinatal period. Their treatment is complicated, resulting in great psychological and financial burden on the child and his family. Recently, several studies have demonstrated that pregnancies conceived with IVF/ICSI have an increased risk of congenital anomalies compared to spontaneous conceptions (3–7), and some meta-analyses have concluded that the pooled risk estimation ranges from 1.32 to 1.37 (8–10).

The underlying mechanisms explaining the association between the risk of congenital anomalies and ART remain unclear, including infertility itself (6) and the increased proportion of multiple births (4, 11). In addition, specific ART procedures, such as ICSI and embryos frozen and thawed, may increase the risk of birth defects (8).

However, few studies have assessed the clinical pregnancy outcomes of fetuses or infants with congenital anomalies born after IVF/ICSI. This study aimed to evaluate the prevalence and prognosis of congenital anomalies among pregnancies conceived through IVF/ICSI treatments from 2010 to 2019, and to explore the factors contributing to poor prognosis.

## Materials and Methods

### Study Design

This retrospective, multicenter, cohort study collected infertile patients undergoing IVF/ICSI cycles at 15 reproductive centers in China. This study was approved by the Medical Science Research Ethics Committee of Peking University Third Hospital (IRB00006761-M2019487). All data collection and analysis procedures conducted in this trial were performed in accordance with the Declaration of Helsinki. Individual informed consent was waived, as it is a retrospective study.

### Patients

**Figure 1:**
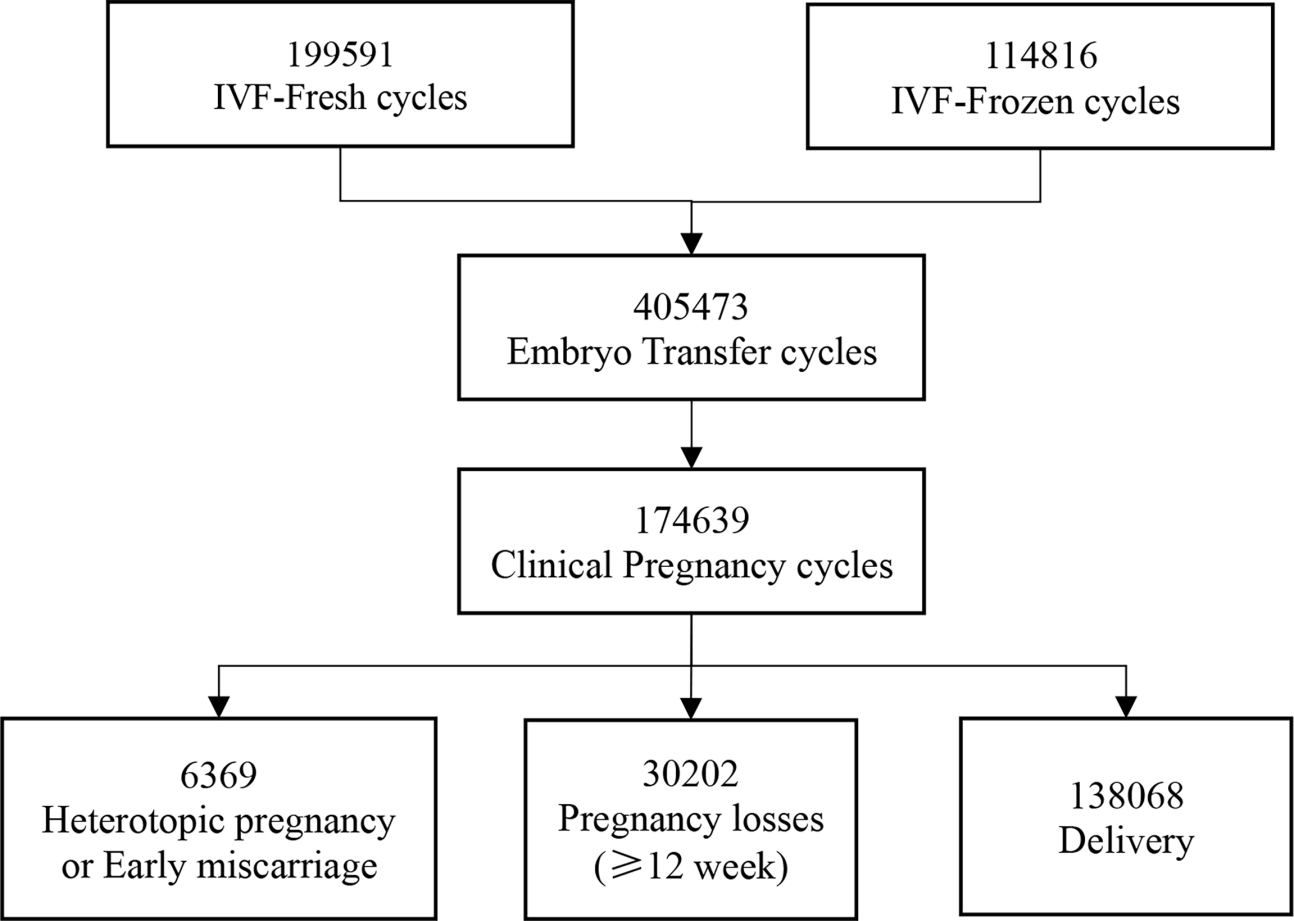
Flowchart showing patient selection.

From January 2010 to December 2019, we followed up 199,591 IVF-fresh cycles and 114,816 IVF-frozen cycles from 15 reproductive centers. Then, a total of 405,473 embryo transfers were performed. Finally, 174,639 women obtained clinical pregnancy, including 6,369 heterotopic pregnancy or early miscarriage and 168,270 intrauterine pregnancy of more than 12 weeks (**Figure 1**).

We collected and recorded detailed information from all centers in this study, including parental basic characteristics (age, body mass index, infertility duration, and parity), IVF/ICSI indications, IVF/ICSI specific techniques (oocyte retrieval protocols, type of fertilization, and fresh or frozen embryos), and IVF/ICSI outcomes (mature oocytes, 2 pro-nuclei [2PN], and fertilized embryos).

Clinical pregnancy outcomes were primarily follow-up data recorded at the end of the pregnancy. Pregnancies ≥28 weeks and <28 weeks but with a live birth were considered deliveries. Pregnancy loss referred to late abortions between 12 and 28 weeks, including spontaneous abortions, embryo damage, and induced abortions for various reasons, divided into miscarriages <20 and ≥20 weeks.

After confirming intrauterine pregnancy, women were scheduled for a systematic prenatal examination in the obstetrics department to identify the presence of deformities or chromosomal abnormalities. In addition to the malformations detected during pregnancy, any structural, functional, and genetic anomalies diagnosed after late abortion or delivery were defined as congenital anomalies (12), which were classified according to the diagnostic codes of the International Classification of Diseases version 10 (ICD-10).

IVF/ICSI cycles with congenital anomalies were grouped into relatively positive prognosis and poor prognosis based on the outcomes of fetuses or neonates. Based on the Manual of Maternal and Child Health Surveillance of China (13), hospital monitoring on birth defects is from 28 weeks of gestation to 7 days after birth. Thus, we defined the relatively positive prognosis as having one or more live births and neonatal survival for more than 7 days. Poor prognosis covered intrauterine deaths, stillbirths, neonatal deaths within 7 days of birth, therapeutic abortions for fetuses with severe fatal and disabling malformations detected by prenatal examination or non-fatal malformations but requested by their parents, and spontaneous abortions after 12 weeks of gestation with chromosomal abnormalities.

### Influencing Factors

To explore the potential factors influencing the prognosis of offspring with congenital anomalies, we made a comparison on the details collected between the relatively positive prognosis group and the poor prognosis group.

Between the two groups, we also compared the rate of fertilization failure, when the number of 2PN embryos accounted for was less than 25% of the number of oocytes collected in one IVF/ICSI cycle, and their pregnancy outcomes, including pregnancy loss rate, delivery rate, live birth rate, birth weight, and birth height.

### Statistical Analysis

All statistical analyses were conducted using SPSS software, version 26.0 for Windows (SPSS, USA). Continuous variables were reported as mean ± standard deviation (SD). The characteristics of two groups were compared using an independent sample *t-*test or the chi-squared test, as appropriate. Given that many factors may affect the prognosis of pregnancies with congenital anomalies, multivariate logistic regression analysis was performed to analyze the potential influencing factors contributing to poor prognosis, with using the odds ratios (ORs) and corresponding 95% confidence intervals (CIs). Statistical significance was defined as a two-sided *p* < 0.05.

## Results

Over the study period, we followed up a total of 168,270 IVF/ICSI cycles with ongoing intrauterine pregnancies and finally enrolled 2,006 pregnancies with congenital anomalies. The overall prevalence of congenital anomalies in the study population was 1.19%, wherein the prevalence of cycles with late pregnancy loss and delivery was 2.37% (716/30,202) and 0.93% (1,290/138,068), respectively. Based on ICD-10 codes, the incidence of congenital anomalies in specific organ systems is shown in **Table 1**. Malformations of the circulatory system were the most common in all pregnancies following IVF/ICSI, with a frequency of 0.34%, while genital malformations were the least common (0.02%). In IVF/ICSI cycles with late abortion, chromosomal abnormality was the main congenital anomaly, accounting for 37.99% of cases (272/716).

**Table 1:** Congenital anomalies from *in vitro* fertilization cycles.

ICD-10 code	Item	Pregnancy losses	Delivery	All*	OR 95% CI
/	No. of cycles (%)	30,202	138,068	168,270	/
/	No. of congenital anomalies (%)	716 (2.37%)	1,290 (0.93%)	2,006 (1.19%)	2.575 (2.348, 2.823)
Q00–Q07	Congenital malformations of the nervous system	69 (0.23%)	96 (0.07%)	165 (0.10%)	3.291 (2.415, 4.485)
Q10–Q18	Congenital malformations of eye, ear, face, and neck	8 (0.03%)	93 (0.07%)	101 (0.06%)	0.393 (0.191, 0.809)
Q20–Q28	Congenital malformations of the circulatory system	134 (0.44%)	440 (0.32%)	574 (0.34%)	1.394 (1.148, 1.692)
Q30–Q34	Congenital malformations of the respiratory system	3 (0.01%)	54 (0.04%)	57 (0.03%)	0.254 (0.079, 0.812)
Q35–Q37	Cleft lip and cleft palate	28 (0.09%)	67 (0.05%)	95 (0.06%)	1.911 (1.229, 2.971)
Q38–Q45	Other congenital malformations of the digestive system	7 (0.02%)	68 (0.05%)	75 (0.04%)	0.470 (0.216, 1.024)
Q50–Q56	Congenital malformations of genital organs	0 (0.00%)	27 (0.02%)	27 (0.02%)	/ (*p* = 0.015)
Q60–Q64	Congenital malformations of the urinary system	18 (0.06%)	86 (0.06%)	104 (0.06%)	0.957 (0.576, 1.590)
Q65–Q79	Congenital malformations and deformations of the musculoskeletal system	41 (0.14%)	156 (0.11%)	197 (0.12%)	1.202 (0.852, 1.696)
Q80–Q89	Other congenital malformations	43 (0.14%)	86 (0.06%)	129 (0.08%)	2.288 (1.586, 3.299)
Q90–Q99	Chromosomal abnormalities, not elsewhere classified	272 (0.90%)	38 (0.03%)	310 (0.18%)	33.010 (23.504, 46.362)
/	Multi-malformations	93 (0.31%)	79 (0.06%)	172 (0.10%)	5.395 (3.996, 7.284)

*Persistent intrauterine pregnancy, intrauterine pregnancy of 12 weeks or more.

Among all IVF/ICSI cycles with congenital anomalies, the rate of relatively positive prognosis was 61.39% (1,258/2,006). Mothers in the relatively positive prognosis group tended to be younger (31.77 ± 4.30 vs. 32.95 ± 4.80, *p* < 0.001); the trend continued for the fathers (33.44 ± 5.27 vs. 34.75 ± 5.93, *p* < 0.001). Furthermore, they were more likely to have primary infertility (57.87% vs. 51.34%, *p* = 0.004) and be nulliparous (89.43% vs. 85.70%, *p* = 0.013) than were those in the poor prognosis group. No statistically significant differences were found in mean body mass index (BMI) of the couples, infertility duration, or IVF/ICSI indications (**Table 2**).

**Table 2:** Basal characteristics of patients with congenital anomalies after *in vitro* fertilization.

	Relatively positive prognosis	Poor prognosis	All	*P*-value
Treatment cycles	1,258	748	2,006	/
Maternal age (years)	31.77 ± 4.30	32.95 ± 4.80	32.21 ± 4.53	<0.001
<30 (%)	401 (31.88%)	194 (25.94%)	595 (29.66%)	<0.001
30–<35 (%)	532 (42.29%)	284 (37.97%)	816 (40.68%)	
35–<40 (%)	274 (21.78%)	195 (26.07%)	469 (23.38%)	
≥40 (%)	51 (4.05%)	75 (10.03%)	126 (6.28%)	
Maternal body mass index (kg/m^2^)	22.39 ± 3.16	22.66 ± 3.52	22.49 ± 3.30	0.115
<18.5 (%)	81 (7.72%)	42 (6.94%)	123 (7.44%)	0.533
18.5–<24.0 (%)	681 (64.92%)	392 (64.79%)	1,073 (64.87%)	
24.0–<28.0 (%)	230 (21.93%)	128 (21.16%)	358 (21.64%)	
≥28.0 (%)	57 (5.43%)	43 (7.11%)	100 (6.05%)	
Unknown (%)	209 (16.61%)	143 (19.12%)	352 (17.55%)	/
Paternal age (years)	33.44 ± 5.27	34.75 ± 5.93	33.93 ± 5.56	<0.001
<30 (%)	287 (22.85%)	151 (20.19%)	438 (21.86%)	<0.001
30–<35 (%)	490 (39.01%)	235 (31.42%)	725 (36.18%)	
35–<40 (%)	335 (26.67%)	208 (27.81%)	543 (27.10%)	
≥40 (%)	144 (11.46%)	154 (20.59%)	298 (14.87%)	
Unknown (%)	2 (0.16%)	0 (0.00%)	2 (0.10%)	/
Paternal body mass index (kg/m^2^)	24.85 ± 3.55	24.75 ± 3.33	24.81 ± 3.47	0.559
<18.5 (%)	27 (2.75%)	14 (2.39%)	41 (2.61%)	0.784
18.5–<24.0 (%)	382 (38.86%)	242 (41.30%)	624 (39.77%)	
24.0–<28.0 (%)	404 (41.10%)	235 (40.10%)	639 (40.73%)	
≥28.0 (%)	170 (17.29%)	95 (16.21%)	265 (16.89%)	
Unknown (%)	275 (21.86%)	162 (21.66%)	437 (21.78%)	/
Infertility duration (years)	4.19 ± 3.21	4.06 ± 3.10	4.14 ± 3.17	0.389
Primary infertility (%)	728 (57.87%)	384 (51.34%)	1,112 (55.43%)	0.004
Nulliparous (%)	1,125 (89.43%)	641 (85.70%)	1,766 (88.04%)	0.013
IVF indications (%)				
Pelvic and tubal disorder	457 (36.33%)	265 (35.43%)	722 (35.99%)	0.455
Ovulatory disorder	98 (7.79%)	58 (7.75%)	156 (7.78%)	
Endometriosis	34 (2.70%)	21 (2.81%)	55 (2.74%)	
Mixed female infertility factors	74 (5.88%)	28 (3.74%)	102 (5.08%)	
Other female infertility factors*	6 (0.48%)	4 (0.53%)	10 (0.50%)	
Oligo-, asthen-, and/or terato-spermia	215 (17.09%)	132 (17.65%)	347 (17.30%)	
Ejaculation disorder	9 (0.72%)	2 (0.27%)	11 (0.55%)	
Azoospermia	43 (3.42%)	32 (4.28%)	75 (3.74%)	
Mixed female and male infertility factors	230 (18.28%)	136 (18.18%)	366 (18.25%)	
Chromosomal abnormality	16 (1.27%)	10 (1.34%)	26 (1.30%)	
Unexplained	76 (6.04%)	60 (8.02%)	136 (6.78%)	

*Other female infertility factors, including uterine malformations and immune infertility.

Of the 2,006 IVF/ICSI cycles, 522 frozen cycles had missing IVF/ICSI laboratory information. There was a statistically significant difference in the method of fertilization between the two study groups. The rate of ICSI was higher in the relatively positive prognosis group (35.93% vs. 29.46%, *p* = 0.011). In cycles treated with ICSI, the rates of mature oocytes (84.41% vs. 87.30%, *p* = 0.002) and 2PN embryos (68.84% vs. 72.90%, *p* = 0.002) were lower in pregnancies with poor prognosis. Furthermore, the rate of fertilization failure (2PN rate <25%) in the poor prognosis group was significantly higher than in the relatively positive prognosis group (10.89% vs. 5.09%, *p* < 0.001) (**Table 3**). In addition, no differences were observed when comparing the oocyte retrieval protocols, fresh or frozen embryo transfer, and the embryo stage at transfer during IVF/ICSI conception between the two groups.

**Table 3:** Characteristics of IVF.

	Relatively positive prognosis	Poor prognosis	All	*p*-value
Treatment cycles	1,258	748	2,006	/
Protocol (%)				
Stimulate protocol	990 (78.70%)	595 (79.55%)	1,585 (79.01%)	0.400
Micro stimulate protocol	117 (9.30%)	57 (7.62%)	174 (8.67%)	
Natural cycle	151 (12.00%)	96 (12.83%)	247 (12.31%)	
ART (%)				
IVF-Fresh cycles	515 (40.94%)	307 (41.04%)	822 (40.98%)	0.181
IVF-Frozen cycles	500 (39.75%)	358 (47.86%)	858 (42.77%)	
PGT	20 (1.59%)	12 (1.60%)	32 (1.60%)	
Fertilization cycles				
IVF-ET (%)	592 (64.07%)	395 (70.54%)	987 (66.51%)	0.011
ICSI (%)	332 (35.93%)	165 (29.46%)	497 (33.49%)	
Unknown (%)	334 (26.55%)	188 (25.13%)	522 (26.02%)	/
IVF (include rescue ICSI) (%)*	592	395	987	/
No. of 2PN embryos/No. of oocytes collected (%)	4,708/7,069 (66.60%)	2,801/4,259 (65.77%)	7,509/11,328 (66.29%)	0.363
No. of fertilization embryos/No. of oocytes collected (%)	5,462/7,069 (77.27%)	3,319/4,259 (77.93%)	8,781/11,328 (77.52%)	0.414
ICSI (include half-ICSI) (%)*	332	165	497	/
No. of mature oocytes/No. of oocytes collected (%)	3,671/4,205 (87.30%)	1,781/2,110 (84.41%)	5,452/6,315 (86.33%)	0.002
No. of 2PN embryos/No. of mature oocytes (%)	2,676/3,671 (72.90%)	1,226/1,781 (68.84%)	3,902/5,452 (71.57%)	0.002
No. of fertilization embryos/No. of mature oocytes (%)	2,921/3,671 (79.57%)	1,361/1,781 (76.42%)	4,282/5,452 (78.54%)	0.008
Fertilization failure cycles (2PN rate < 25%) (%)*	47 (5.09%)	61 (10.89%)	108 (7.28%)	<0.001
Embryos transferred (%)				
1	233 (18.52%)	192 (25.63%)	425 (21.19%)	<0.001
2	951 (75.60%)	507 (67.69%)	1,458 (72.68%)	
3	74 (5.88%)	49 (6.54%)	123 (6.13%)	
Embryo stage at transfer (%)				
Cleavage stage	825 (65.58%)	484 (64.62%)	1,309 (65.25%)	0.691
Blastocyst stage	433 (34.42%)	264 (35.25%)	697 (34.75%)	

*Out of the 2,006 IVF cycles, IVF laboratory information was collected for 1,484 cycles (73.98%).

2PN, 2 pro-nuclei; ICSI, intracytoplasmic sperm injection; IVF, in vitro fertilization; PGT, preimplantation genetic testing.

The clinical outcomes of the study populations were demonstrated in **Table 4**. Newborns with relatively positive prognosis yielded a significantly superior birth weight (2,616.06 ± 754.53 g vs. 1,936.07 ± 1,186.61 g, *p* = 0.001) and birth height (47.25 ± 4.29 cm vs. 40.33 ± 7.58 cm, *p* < 0.001).

**Table 4:** Clinical outcomes.

	Relatively positive prognosis	Poor prognosis	All	*P*-value
Treatment cycles	1258	748	2006	/
Pregnancy outcomes				
Pregnancy losses (weeks)	0 (0.00%)	665 (88.90%)	665 (33.15%)	<0.001
12–<20 (%)	0 (0.00%)	204 (27.27%)	204 (10.17%)	/
20–<28 (%)	0 (0.00%)	461 (61.63%)	461 (22.98%)	
Delivery (weeks)*	1,258 (100.00%)	83 (11.10%)	1,341 (66.85%)	<0.001
<28 (%)	37 (2.94%)	1 (0.13%)	38 (1.89%)	/
28–<34 (%)	197 (15.66%)	60 (8.02%)	257 (12.81%)	
34–<37 (%)	308 (24.48%)	8 (1.07%)	316 (15.75%)	
≥37 (%)	716 (56.92%)	14 (1.87%)	730 (36.39%)	
Live births** (%)	1,258 (100.00%)	18 (2.41%)	1,276 (63.61%)	<0.001
Live birth infants	1,749	22	1,771	/
Congenital anomalies	1,305 (74.61%)	21 (95.45%)	1,326 (74.87%)	0.025
Healthy	444 (25.39%)	1 (4.55%)***	445 (25.13%)	
Live birth weight (g)	2,616.06 ± 754.53	1,936.07 ± 1186.61	2,610.48 ± 761.01	0.001
<1,500 (%)	132 (7.55%)	7 (31.82%)	139 (7.85%)	<0.001
1,500–<2,500 (%)	544 (31.10%)	2 (9.09%)	546 (30.83%)	
2,500–<4,500 (%)	1,004 (57.40%)	5 (22.73%)	1,009 (56.97%)	
≥4,500 (%)	10 (0.57%)	0 (0.00%)	10 (0.56%)	
Unknown	59 (3.37%)	8 (36.36%)	67 (3.78%)	/
Live birth height (cm)	47.25 ± 4.29	40.33 ± 7.58	47.19 ± 4.37	<0.001
Unknown	677 (38.71%)	13 (59.09%)	690 (38.96%)	/

*Number of deliveries at or above 28 weeks gestation and deliveries under 28 weeks but with live births.

**Number of cycles having one or more live births.

***Newborn died with no congenital anomalies.

Multivariate logistic regression analysis showed that pluriparity, early preterm birth (delivery at 28–34 weeks), and fertilization failure were factors for poor prognosis in IVF/ICSI pregnancies with congenital anomalies after adjusting for all confounding variables (**Table 5**). The resulting adjusted OR for fertilization failure was 0.180 (95% CI: 0.061–0.528, *p* = 0.002) for patients with a relatively positive prognosis.

**Table 5:** Logistic regression of the relatively positive prognosis rate in IVF cycles.

	*B*	*P*-value	OR	95% CI for OR	
				Lower	Upper
Maternal age (years)					
<30		0.120			
30–<35	0.307	0.511	1.359	0.545	3.391
35–<40	1.145	0.098	3.141	0.808	12.208
≥40	3.693	0.032	40.158	1.382	1167.321
Paternal age (years)					
<30		0.241			
30–<35	0.502	0.295	1.653	0.645	4.232
35–<40	−0.390	0.499	0.677	0.219	2.098
≥40	−0.475	0.555	0.622	0.128	3.009
Primary infertility	−0.100	0.794	0.904	0.426	1.920
Nulliparous	1.420	0.007	4.138	1.478	11.581
ART					
IVF-Fresh cycles		0.808			
IVF-Frozen cycles	0.225	0.514	1.253	0.637	2.465
PGT	15.323	0.996	4,515,108	0.000	.
Pregnancy outcomes (weeks)					
Delivery, ≥37		0.000			
Pregnancy losses, 12–<20	−36.392	0.990	0.000	0.000	.
Pregnancy losses, 20–<28	−36.846	0.986	0.000	0.000	.
Delivery, <28	17.039	0.998	25,118,306	0.000	.
Delivery, 28–<34	−2.657	0.000	0.070	0.030	0.163
Delivery, 34–<37	−0.625	0.226	0.535	0.194	1.473
Congenital anomalies					
Chromosomal abnormalities, not elsewhere classified		0.004			
Congenital malformations of the nervous system	1.521	0.021	4.575	1.253	16.709
Congenital malformations of eye, ear, face, and neck	3.380	0.004	29.363	3.037	283.891
Congenital malformations of the circulatory system	2.418	0.000	11.228	3.631	34.715
Congenital malformations of the respiratory system	3.180	0.006	24.037	2.451	235.762
Cleft lip and cleft palate	1.613	0.079	5.016	0.829	30.334
Other congenital malformations of the digestive system	2.113	0.019	8.271	1.420	48.187
Congenital malformations of genital organs	20.081	0.998	525,983,590	0.000	.
Congenital malformations of the urinary system	17.320	0.990	33,278,477	0.000	.
Congenital malformations and deformations of the musculoskeletal system	2.605	0.001	13.536	3.085	59.390
Other congenital malformations	1.212	0.054	3.360	0.980	11.512
Multi-malformations	2.504	0.001	12.228	2.779	53.808
ICSI	0.540	0.161	1.716	0.807	3.649
Fertilization failure cycles (2PN rate < 25%)	−1.716	0.002	0.180	0.061	0.528
Embryos transferred					
1		0.931			
2	−0.180	0.750	0.836	0.278	2.516
3	−0.002	0.999	0.998	0.160	6.239
Constant	1.768	0.999	5.859		

OR, odds ratio; CI, confidence interval; 2PN, 2 pro-nuclei; ICSI, intracytoplasmic sperm injection; IVF, in vitro fertilization; PGT, preimplantation genetic testing.

## Discussion

In this 2010–2019 multicenter comprehensive follow-up study in China, the overall prevalence of congenital anomalies among intrauterine pregnancies ≥12 weeks conceived through IVF/ICSI was 1.19%. Nearly two-thirds could obtain a relatively positive prognosis with one or more live births surviving more than 7 days. Moreover, patients with fertilization failure were more likely to have a poor prognosis for fetuses with congenital anomalies.

The prevalence of congenital anomalies in the present study was similar to the rate of 1.23% observed in a multicenter study conducted from 2004 to 2008 (14). Another single-center study indicated that congenital anomalies among infants conceived using ART ranged from 1.10% to 1.20% (15). However, both studies were limited to live births, and other pregnancy outcomes, such as stillbirths or therapeutic labor induction, were not considered. In contrast, our study showed a lower prevalence of birth defects (0.93%) in cycles that resulted in delivery.

Consistent with previous studies (5, 15), the most common anomalies were malformations of the circulatory system in pregnancies after IVF/ICSI. Chromosomal and musculoskeletal anomalies followed behind. The incidence of chromosomal abnormalities in patients who experienced abortion was 33 times higher than in women who gave birth (odds ratio [OR] = 33.010, 95% CI = 23.504, 46.362). Infertile patients may have underlying genetic anomalies, and some chromosomal abnormalities and genetic pathogenic variants could lead to infertility (16). The genetic defects might transmit to offspring, associated with congenital anomalies (17). In the process of IVF/ICSI treatment, preimplantation genetic testing (PGT) or single-nucleotide polymorphism (SNP) technology can effectively reduce the prevalence of recurrent pregnancy loss or chromosomal karyotype abnormalities (18, 19). Therefore, a PGT biopsy should be performed in patients with indications. Among the infants born, circulatory malformations remain the most common birth defects, followed by musculoskeletal deformities, while chromosomal abnormalities were the rarest after genital malformations. This finding was different from the results of Han et al., in which gastrointestinal anomalies were the second and cheilopalatognathus was the third.

To date, several studies have indicated that ART use is associated with an increased risk of congenital anomalies (2–7, 11, 20, 21). Levi et al. enrolled pregnancies of more than 12 weeks and early spontaneous abortions and ectopic pregnancies (3). They reported a prevalence of 3.8% for congenital anomalies after ART, which was significantly higher than the general population (2.05%). Shechter et al. only included live births and found that newborns conceived with ART were more likely to have birth defects compared with those conceived without ART in the US (OR = 2.14, 95% CI = 1.94, 2.35) (7). Another recent research on offspring obtained through ART or non-ART in Beijing showed a higher rate of birth defects in ART offspring (crude RR = 1.49; 95% CI = 1.26, 1.76) (2). A meta-analysis by Hansen et al. (8) reviewed 45 cohort studies and identified a pooled relative risk estimation of 1.32 (95% CI = 1.24, 1.42). The observed increased incidence of congenital anomalies may be explained by advanced parental age (22, 23), multiple pregnancies (4, 11), and underlying causes of infertility (3, 6, 24) among infertile patients undergoing ART treatments.

Few studies have explored the prognosis of fetuses with congenital anomalies. Our study showed that the relatively positive prognosis rate was 61.39% (1,258/2,006). Previously, Zhang et al. recorded the outcomes of fetuses with congenital heart disease (25). In this study, less than one in five newborns was born alive (346/1,851), of whom 34 died within 7 days after birth. They indicated that gestational age at delivery was the only risk factor contributing to neonatal death in the first week of life (*p* < 0.001).

The present study found statistically significant differences between female sex, male mean age, and delivery history (nulliparous or not) between the two prognosis groups. Older women have a lower quality of oocytes and an increased risk of chromosomal abnormalities in their offspring (26), resulting in a poor prognosis for fetuses with congenital anomalies. Primary infertility and nulliparity were more common in the relatively positive prognosis group. After controlling for other underlying influencing factors, nulliparity still showed a relatively positive outcome. The hypothesis was that a history of abnormal gestation and birth might impact pregnancy outcomes in infertile patients undergoing IVF/ICSI cycles.

It has been proposed that specific ART procedures, such as fresh or frozen embryo transfer after IVF/ICSI or ICSI, have little impact on the prevalence of congenital anomalies (27, 28). A 2012 meta-analysis did not find any difference in risk between the two insemination methods (10). Furthermore, in our study, although more ICSI cycles were observed in the relatively positive prognosis group (35.93% vs. 29.46%, *p* = 0.011), there was no substantial difference in the effect on prognosis whether patients were treated with ICSI (*p* = 0.161) after excluding other confounding factors. When comparing fresh and frozen-thawed embryo transfers, our study showed a similar rate of relatively positive prognosis (*p* = 0.808). The consensus is that the transfer of a single embryo has better perinatal outcomes for both mothers and offspring (29, 30). In contrast to previous reports, our study found that single-embryo transfer was associated with a lower chance of favorable prognosis; however, the multivariate logistic regression analysis identified that the number of transferred embryos had no significant effect on prognosis (*p* = 0.931).

More importantly, pregnancies with a poor prognosis had a lower rate of mature oocytes and a higher fertilization failure rate (2PN rate < 25%). Multivariate logistic regression analysis showed that the relatively positive prognosis rate of fertilization failure cycles was 0.180 times that of normal fertilization cycles. Epigenetic remodeling, including DNA methylation, chromatin accessibility, and histone modifications, occurs primarily during human gametogenesis and early embryonic development (31). ART procedures may affect epigenetic reprogramming processes, causing severe defects in offspring (32, 33). Additionally, several genes reportedly cause oocyte maturation arrest, fertilization failure, embryonic arrest, and preimplantation embryonic lethality (34). Combined with the results of the current study, infertility patients with fewer mature oocytes and more failed fertilization have a plausible increased risk of abnormal gametes, consequently leading to poor quality embryos. Poor quality day 5 embryos transferred were more likely to have major anomalies and chromosomal abnormalities (35) and had a further poor prognosis.

Several limitations exist in our study. First, there are no clear guidelines for defining favorable or unfavorable prognosis. In this study, we defined a relatively positive prognosis as having one or more live births surviving for more than 7 days. However, quite a few babies suffering from congenital anomalies die within the first month of life. At present, in majority of reproductive centers in China, the routine follow-up endpoint for ongoing pregnancies conceived by IVF/ICSI is 1–2 weeks after the expected date of confinement. The terminal point of the Chinese hospital-based birth defect surveillance system is 7 days after birth (35). As a multicenter study, it was difficult to obtain the outcomes of newborns aged 1 month age or older. Second, we did not compare the clinical outcomes of fetuses or infants with congenital anomalies after IVF/ICSI with those conceived naturally, as the participants were recruited from centers for reproductive medicine. Further research is needed to compare the prognosis of IVF/ICSI and spontaneous conceptions with congenital anomalies. Third, some laboratory data for fresh oocyte retrieval cycles corresponding to frozen-thawed embryo transfer cycles were unavailable, because some participating centers had limited electronic medical record systems. However, these deletions were completely random and did not significantly influence the results of our study.

In conclusion, this is the first multicenter study on the prognosis of pregnancies with congenital anomalies after IVF/ICSI. Moreover, our study suggests that poor fertilization rates during IVF treatment are associated with a poor prognosis in fetuses or neonates with congenital anomalies. Hence, couples experiencing fertilization failure should be recommended to strengthen obstetric management and active prenatal diagnosis.

## Data Availability Statement

The datasets presented in this article are not readily available because:

According to the requirements of this research funding, the original data are uniformly managed by the database of the Information Center for the National Health Commission of the People’s Republic of China and cannot be freely shared.

Requests to access the datasets should be directed to:

Wang Yuanyuan, yyuanwang@163.com.

## Ethics Statement

The studies involving human participants were reviewed and approved by Peking University Third Hospital Medical Science Research Ethics Committee. Written informed consent for participation was not required for this study in accordance with the national legislation and the institutional requirements.

## Author Contributions

HC, YW, and JQ contributed to conception and design of the study. YH, HW, XH, CL, and XJ were in charge of data collection. JB and LC performed the statistical analysis and wrote the first draft of the manuscript. All authors contributed to manuscript revision, read, and approved the submitted version. All authors contributed to the article and approved the submitted version.

## Funding

This work was supported by the National Key Research and Development Program of China (No. 2019YFC1005106).

## Conflict of Interest

The authors declare that the research was conducted in the absence of any commercial or financial relationships that could be construed as a potential conflict of interest.

## Publisher’s Note

All claims expressed in this article are solely those of the authors and do not necessarily represent those of their affiliated organizations, or those of the publisher, the editors and the reviewers. Any product that may be evaluated in this article, or claim that may be made by its manufacturer, is not guaranteed or endorsed by the publisher.

## References

[1] Iacusso C Iacobelli BD Morini F Totonelli G Viggiano M Caforio L. Assisted Reproductive Technology and Anorectal Malformation: A Single-Center Experience. Front Pediatr (2021) 9:705385. doi: 10.3389/fped.2021.705385 34604138 PMC8482871

[2] Zhang L Zhang W Xu H Liu K. Birth Defects Surveillance After Assisted Reproductive Technology in Beijing: A Whole of Population-Based Cohort Study. BMJ Open (2021) 11(6):e044385. doi: 10.1136/bmjopen-2020-044385 PMC8231031 34162637

[3] Levi Setti PE Moioli M Smeraldi A Cesaratto E Menduni F Livio S. Obstetric Outcome and Incidence of Congenital Anomalies in 2351 IVF/ICSI Babies. J Assisted Reprod Genet (2016) 33(6):711–7. doi: 10.1007/s10815-016-0714-4 PMC4889486 27116010

[4] Wen SW Miao Q Taljaard M Lougheed J Gaudet L Davies M. Associations of Assisted Reproductive Technology and Twin Pregnancy With Risk of Congenital Heart Defects. JAMA Pediatr (2020) 174(5):446–54. doi: 10.1001/jamapediatrics.2019.6096 PMC7042937 32091547

[5] Galdini A Fesslova VME Gaeta G Candiani M Pozzoni M Chiarello C. Prevalence of Congenital Heart Defects in Pregnancies Conceived by Assisted Reproductive Technology: A Cohort Study. J Clin Med (2021) 10(22):5363. doi: 10.3390/jcm10225363 PMC8621349 34830645

[6] Seggers J de Walle HE Bergman JE Groen H Hadders-Algra M Bos ME. Congenital Anomalies in Offspring of Subfertile Couples: A Registry-Based Study in the Northern Netherlands. Fertil Steril (2015) 103(4):1001–10.e3. doi: 10.1016/j.fertnstert.2014.12.113 25624190

[7] Shechter-Maor G Czuzoj-Shulman N Spence AR Abenhaim HA. The Effect of Assisted Reproductive Technology on the Incidence of Birth Defects Among Livebirths. Arch Gynecol Obstetrics (2018) 297(6):1397–403. doi: 10.1007/s00404-018-4694-8 29450693

[8] Hansen M Kurinczuk JJ Milne E de Klerk N Bower C. Assisted Reproductive Technology and Birth Defects: A Systematic Review and Meta-Analysis. Hum Reprod Update (2013) 19(4):330–53. doi: 10.1093/humupd/dmt006 23449641

[9] Qin J Sheng X Wang H Liang D Tan H Xia J. Assisted Reproductive Technology and Risk of Congenital Malformations: A Meta-Analysis Based on Cohort Studies. Arch Gynecol Obstetrics (2015) 292(4):777–98. doi: 10.1007/s00404-015-3707-0 25877221

[10] Wen J Jiang J Ding C Dai J Liu Y Xia Y. Birth Defects in Children Conceived by *In Vitro* Fertilization and Intracytoplasmic Sperm Injection: A Meta-Analysis. Fertil Steril (2012) 97(6):1331–7.e1-4. doi: 10.1016/j.fertnstert.2012.02.053 22480819

[11] Liberman RF Getz KD Heinke D Luke B Stern JE Declercq ER. Assisted Reproductive Technology and Birth Defects: Effects of Subfertility and Multiple Births. Birth Defects Res (2017) 109(14):1144–53. doi: 10.1002/bdr2.1055 PMC5555800 28635008

[12] Zegers-Hochschild F Adamson GD de Mouzon J Ishihara O Mansour R Nygren K. International Committee for Monitoring Assisted Reproductive Technology (ICMART) and the World Health Organization (WHO) Revised Glossary of ART Terminology, 2009. Fertil Steril (2009) 92(5):1520–4. doi: 10.1016/j.fertnstert.2009.09.009 19828144

[13] National Health and Family Planning Commission. The Manual of Maternal and Child Health Surveillance of China (2018). Available at: http://www.mchscn.cn/National-22/450.html (Accessed August 30, 2018).

[14] Yan J Huang G Sun Y Zhao X Chen S Zou S. Birth Defects After Assisted Reproductive Technologies in China: Analysis of 15,405 Offspring in Seven Centers (2004 to 2008). Fertil Sterility (2011) 95(1):458–60. doi: 10.1016/j.fertnstert.2010.08.024 20850722

[15] Han Y Luo H Zhang Y. Congenital Anomalies in Infants Conceived by Infertile Women Through Assisted Reproductive Technology: A Cohort Study 2004-2014. Exp Ther Med (2018) 16(4):3179–85. doi: 10.3892/etm.2018.6572 PMC6125944 30214541

[16] Wu J Li D Liu X Li Q He X Wei J. IDDB: A Comprehensive Resource Featuring Genes, Variants and Characteristics Associated With Infertility. Nucleic Acids Res (2021) 49(D1):D1218–24. doi: 10.1093/nar/gkaa753 PMC7779019 32941628

[17] Buckett WM Tan SL. Congenital Abnormalities in Children Born After Assisted Reproductive Techniques: How Much Is Associated With the Presence of Infertility and How Much With its Treatment? Fertil Sterility (2005) 84(5):1318–9; discussion 27. doi: 10.1016/j.fertnstert.2005.04.065 16275221

[18] Kimelman D Pavone ME. Non-Invasive Prenatal Testing in the Context of IVF and PGT-A. Best Pract Res Clin Obstetrics Gynaecol (2021) 70:51–62. doi: 10.1016/j.bpobgyn.2020.07.004 32739290

[19] Rajcan-Separovic E. Next Generation Sequencing in Recurrent Pregnancy Loss-Approaches and Outcomes. Eur J Med Genet (2020) 63(2):103644. doi: 10.1016/j.ejmg.2019.04.001 30991114

[20] Boulet SL Kirby RS Reefhuis J Zhang Y Sunderam S Cohen B. Assisted Reproductive Technology and Birth Defects Among Liveborn Infants in Florida, Massachusetts, and Michigan, 2000-2010. JAMA Pediatr (2016) 170(6):e154934. doi: 10.1001/jamapediatrics.2015.4934 27043648 PMC4899282

[21] Giorgione V Parazzini F Fesslova V Cipriani S Candiani M Inversetti A. Congenital Heart Defects in IVF/ICSI Pregnancy: Systematic Review and Meta-Analysis. Ultrasound Obstetrics Gynecol Off J Int Soc Ultrasound Obstetrics Gynecol (2018) 51(1):33–42. doi: 10.1002/uog.18932 29164811

[22] Frederiksen LE Ernst A Brix N Braskhøj Lauridsen LL Roos L Ramlau-Hansen CH. Risk of Adverse Pregnancy Outcomes at Advanced Maternal Age. Obstetrics Gynecol (2018) 131(3):457–63. doi: 10.1097/aog.0000000000002504 29420406

[23] Lean SC Derricott H Jones RL Heazell AEP. Advanced Maternal Age and Adverse Pregnancy Outcomes: A Systematic Review and Meta-Analysis. PloS One (2017) 12(10):e0186287. doi: 10.1371/journal.pone.0186287 29040334 PMC5645107

[24] Davies MJ Moore VM Willson KJ Van Essen P Priest K Scott H. Reproductive Technologies and the Risk of Birth Defects. New Engl J Med (2012) 366(19):1803–13. doi: 10.1056/NEJMoa1008095 22559061

[25] Zhang W Xu HY Zhang YC Liu KB. Delayed Diagnosis of Critical Congenital Heart Defects Predicting Risk Factors and Survival Rate in Newborns in Beijing: A Retrospective Study. J Int Med Res (2021) 49(7):3000605211028028. doi: 10.1177/03000605211028028 34264137 PMC8287373

[26] Mikwar M MacFarlane AJ Marchetti F. Mechanisms of Oocyte Aneuploidy Associated With Advanced Maternal Age. Mutat Res Rev Mutat Res (2020) 785:108320. doi: 10.1016/j.mrrev.2020.108320 32800274

[27] Beltran Anzola A Pauly V Montjean D Meddeb L Geoffroy-Siraudin C Sambuc R. No Difference in Congenital Anomalies Prevalence Irrespective of Insemination Methods and Freezing Procedure: Cohort Study Over Fourteen Years of an ART Population in the South of France. J Assisted Reprod Genet (2017) 34(7):867–76. doi: 10.1007/s10815-017-0903-9 PMC5476536 28444613

[28] Maheshwari A Pandey S Shetty A Hamilton M Bhattacharya S. Obstetric and Perinatal Outcomes in Singleton Pregnancies Resulting From the Transfer of Frozen Thawed Versus Fresh Embryos Generated Through *In Vitro* Fertilization Treatment: A Systematic Review and Meta-Analysis. Fertil Sterility (2012) 98(2):368–77.e1-9. doi: 10.1016/j.fertnstert.2012.05.019 22698643

[29] Grady R Alavi N Vale R Khandwala M McDonald SD. Elective Single Embryo Transfer and Perinatal Outcomes: A Systematic Review and Meta-Analysis. Fertil Steril (2012) 97(2):324–31. doi: 10.1016/j.fertnstert.2011.11.033 22177461

[30] Kissin DM Kulkarni AD Kushnir VA Jamieson DJ. Number of Embryos Transferred After *In Vitro* Fertilization and Good Perinatal Outcome. Obstetrics Gynecol (2014) 123(2 Pt 1):239–47. doi: 10.1097/aog.0000000000000106 PMC4607031 24402601

[31] Wang Y Liu Q Tang F Yan L Qiao J. Epigenetic Regulation and Risk Factors During the Development of Human Gametes and Early Embryos. Annu Rev Genomics Hum Genet (2019) 20:21–40. doi: 10.1146/annurev-genom-083118-015143 30917080

[32] Uyar A Seli E. The Impact of Assisted Reproductive Technologies on Genomic Imprinting and Imprinting Disorders. Curr Opin Obstetrics Gynecol (2014) 26(3):210–21. doi: 10.1097/gco.0000000000000071 PMC4123998 24752003

[33] Hattori H Hiura H Kitamura A Miyauchi N Kobayashi N Takahashi S. Association of Four Imprinting Disorders and ART. Clin Epigenet (2019) 11(1):21. doi: 10.1186/s13148-019-0623-3 PMC6367766 30732658

[34] Sang Q Zhou Z Mu J Wang L. Genetic Factors as Potential Molecular Markers of Human Oocyte and Embryo Quality. J Assisted Reprod Genet (2021) 38(5):993–1002. doi: 10.1007/s10815-021-02196-z PMC8190202 33895934

[35] Abel K Healey M Finch S Osianlis T Vollenhoven B. Associations Between Embryo Grading and Congenital Malformations in IVF/ICSI Pregnancies. Reprod Biomed Online (2019) 39(6):981–9. doi: 10.1016/j.rbmo.2019.07.035 31606300

